# A Simple Screening Approach To Prioritize Genes for Functional Analysis Identifies a Role for Interferon Regulatory Factor 7 in the Control of Respiratory Syncytial Virus Disease

**DOI:** 10.1128/mSystems.00051-16

**Published:** 2016-06-28

**Authors:** Jacqueline U. McDonald, Myrsini Kaforou, Simon Clare, Christine Hale, Maria Ivanova, Derek Huntley, Marcus Dorner, Victoria J. Wright, Michael Levin, Federico Martinon-Torres, Jethro A. Herberg, John S. Tregoning

**Affiliations:** a Mucosal Infection and Immunity Group, Section of Virology, Imperial College London, St. Mary’s Campus, London, United Kingdom; b Section of Paediatrics, Imperial College London, St. Mary’s Campus, London, United Kingdom; c Wellcome Trust Sanger Institute, Wellcome Trust Genome Campus, Hinxton, United Kingdom; d Imperial College Centre for Integrative Systems Biology and Bioinformatics, Imperial College London, London, United Kingdom; e Molecular Virology, Section of Virology, Imperial College London, St. Mary’s Campus, London, United Kingdom; f Department of Paediatrics, Hospital Clínico Universitario de Santiago, Santiago de Compostela, Spain; University of California, San Francisco

**Keywords:** host response, immunity, respiratory syncytial virus, viral immunity

## Abstract

Greater understanding of the functions of host gene products in response to infection is required. While many of these genes enable pathogen clearance, some enhance pathogen growth or contribute to disease symptoms. Many studies have profiled transcriptomic and proteomic responses to infection, generating large data sets, but selecting targets for further study is challenging. Here we propose a novel data-mining approach combining multiple heterogeneous data sets to prioritize genes for further study by using respiratory syncytial virus (RSV) infection as a model pathogen with a significant health care impact. The assumption was that the more frequently a gene is detected across multiple studies, the more important its role is. A literature search was performed to find data sets of genes and proteins that change after RSV infection. The data sets were standardized, collated into a single database, and then panned to determine which genes occurred in multiple data sets, generating a candidate gene list. This candidate gene list was validated by using both a clinical cohort and *in vitro* screening. We identified several genes that were frequently expressed following RSV infection with no assigned function in RSV control, including *IFI27*, *IFIT3*, *IFI44L*, *GBP1*, *OAS3*, *IFI44*, and *IRF7*. Drilling down into the function of these genes, we demonstrate a role in disease for the gene for interferon regulatory factor 7, which was highly ranked on the list, but not for *IRF1*, which was not. Thus, we have developed and validated an approach for collating published data sets into a manageable list of candidates, identifying novel targets for future analysis.

**IMPORTANCE** Making the most of “big data” is one of the core challenges of current biology. There is a large array of heterogeneous data sets of host gene responses to infection, but these data sets do not inform us about gene function and require specialized skill sets and training for their utilization. Here we describe an approach that combines and simplifies these data sets, distilling this information into a single list of genes commonly upregulated in response to infection with RSV as a model pathogen. Many of the genes on the list have unknown functions in RSV disease. We validated the gene list with new clinical, *in vitro*, and *in vivo* data. This approach allows the rapid selection of genes of interest for further, more-detailed studies, thus reducing time and costs. Furthermore, the approach is simple to use and widely applicable to a range of diseases.

## Abstract (precis)

Making the most of “big data” is one of the core challenges of current biology. There is a large array of heterogeneous data sets of host gene responses to infection, but these data sets do not inform us about gene function and require specialized skill sets and training for their utilization. Here we describe an approach that combines and simplifies these data sets, distilling this information into a single list of genes commonly upregulated in response to infection with RSV as a model pathogen. Many of the genes on the list have unknown functions in RSV disease. We validated the gene list with new clinical, *in vitro*, and *in vivo* data. This approach allows the rapid selection of genes of interest for further, more-detailed studies, thus reducing time and costs. Furthermore, the approach is simple to use and widely applicable to a range of diseases.

## INTRODUCTION

The interpretation of large data sets—big data—is one of the challenges of modern biology (1). Several powerful approaches have been developed to derive functional correlates from these large data sets, but they all have some limitations. Analysis of correlated or functionally related groups of genes *en bloc* simplifies analysis (2, 3); however, this approach loses gene level detail, particularly for genes with unknown roles. Systems biology approaches to identify key genes within pathways have been applied in vaccination (4) and infection (5) studies, but there is still a requirement to select individual genes for further analysis. High-throughput screenings enable the rapid identification of gene functions in an *in vitro* context (6–8), but these screenings only investigate the role of genes in the context of individual cells, not in relation to the system as a whole. Recently, programs of work have been developed to systematically target every gene in the mouse genome to define its function (9, 10); nevertheless, cost and ethical considerations require a focused selection of the targets of interest. The plethora of data available makes the prioritization of genes for further analysis challenging and often requires specialized skill sets and costly software. We propose a novel and simple approach to integrate published data sets to rapidly identify genes for their functions in the control of infection by using freely available software.

We used this novel approach to identify genes involved in the host response to respiratory syncytial virus (RSV) infection as a proof of principle. RSV is a ubiquitous infection in early life and a significant cause of disease (11). While the majority of children are infected with RSV during infancy, only a small proportion (2%) require hospitalization, of whom many have known risk factors, including prematurity, congenital heart disease, or immunodeficiency. However, the majority of hospitalized children (73 to 85%) have no known risk factor (12, 13). This phenotypic variability in host response may reflect the role of host genetic polymorphisms in protection against or potentiation of severe disease (14). Severe RSV disease is associated with perturbation of normal airway function in the lower respiratory tract, but the events leading to the perturbation of airway function after RSV infection are not clear. Indeed, the cause of disease may be heterogeneous, with virus-induced cell death causing disease in some infants and excess local inflammation having a role in others. The broader cellular immune response to RSV has been well dissected, with protective and pathogenic roles assigned to many cell types, including macrophages (15, 16) and NK (17, 18) and T cells (19, 20), but the molecular immune profile has not been fully explored. A number of studies in the last decade have published host omics profiles of RSV disease, identifying signatures of RSV infection, which enable discrimination between RSV and other respiratory viral infections (21, 22). Published associations of genes with the response to RSV are derived from a diverse range of systems, both *in vitro* and *in vivo*, in humans and mice, and based on RNA, DNA, and protein data (see Table S1 in the supplemental material). However, while these studies have identified genes that change in response to infection, they have not defined the functional roles of individual genes in RSV disease.

10.1128/mSystems.00051-16.2 Table S1  Data sets mined for study. Papers published prior to July 2015 were selected by using the search terms “RSV AND Microarray OR transcriptome OR genetic or proteome” in PubMed. Incorporated are subjective weighting scores based on the types of studies from which they were collated, as follows: human genetic studies, 4; human *in vivo* transcriptomic studies, 3; human *in vitro* studies, 2; murine studies, 1. The sample type, time point, and analysis method are also included. Download Table S1, DOCX file, 0.1 MB. Copyright © 2016 McDonald et al. 2016 McDonald et al. This content is distributed under the terms of the Creative Commons Attribution 4.0 International license.

The aims of the present study were to integrate multiple published data sets to prioritize the genes associated with RSV infection and to dissect their functions in RSV disease. To achieve these goals, we employed a combination of *in silico*, *in vitro*, and *in vivo* approaches. Our conclusions were consistently supported by a new clinical study. Integrating multiple studies in this fashion increases confidence in the role of the genes identified. Utilizing this approach we identified *IRF7* as a key gene in the control of RSV. Here, we demonstrate that integration of published omic data sets with high-throughput studies generates insights into the genetic control of infection.

## RESULTS

### Meta-analysis of RSV data sets reveals functional pathways in the control of RSV.

**FIG 1:**
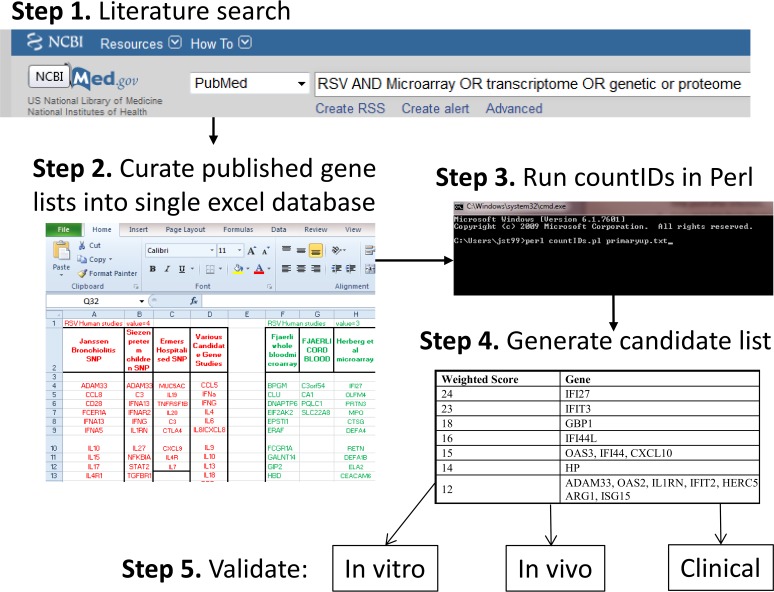
Flowchart of the gene selection method used in this study.

We developed a novel approach to mine the mass of published gene and protein profile data in order to prioritize genes for functional profiling (Fig. 1). A literature search was performed to identify studies using omics tools to analyze the response to RSV infection. Data were collected from multiple published studies of RSV disease, selecting studies that included accessible lists of genes and/or proteins that were detectable following RSV infection of either humans or mice (see Table S1 in the supplemental material) or identified as significant in genetic studies or genome-wide association studies. Because of the heterogeneity of the approaches, when multiple data sets were available in a single study, we focused on the 24 h after infection time points in primary infection (rather than reinfection) and set an arbitrary cutoff of a 2-fold increase or decrease in gene expression for transcriptomic studies. Studies were collated in a single database, and we then used a custom Perl script (countIDs; https://sourceforge.net/projects/countids/) to parse the file to find genes that were present in multiple studies. Genes were ranked by frequency of occurrence and weighted on the basis of the types of studies they appeared in: genetic association studies, *ex vivo* human studies, or *in vitro* human or mouse studies, with more weighting on the human than the murine studies. This subjective weighting score was based on perceived relevance to human infection. Weighting reflected the nature of the input study, not specific data layers, which were treated equally; genes, mRNA, and proteins were given equal weight. A list of candidate genes was then generated (Table 1). Using the weighted analysis, the genes that were most commonly reported as being upregulated were *IFI27*, *IFIT3*, *GBP1*, *IFI44L*, *OAS3*, *IFI44*, and *CXCL10* (Table 1). No functional roles in the control of RSV infection have been previously described for these genes. Fewer genes were downregulated after RSV infection, and they were less uniformly represented between studies; the downregulated genes included *CLC*, *NDUFS1*, and *PFDN5* (Table 2). For comparison, an unweighted analysis was also performed (see Tables S2 and S3 in the supplemental material); this analysis identified similar patterns of genes, with interferon the gene for (IFN) regulatory factor 7 (*IRF7*) relatively higher in the unweighted analyses than in the weighted analyses. Thus, we can take published data sets and condense them into a single list. Interestingly despite the heterogeneous nature of the input studies, we identified a large overlap in the genes identified.

**TABLE 1:** Genes most frequently upregulated following RSV infection^a^

Weighted score	Gene(s)
24	*IFI27*
23	*IFIT3*
18	*GBP1*
16	*IFI44L*
15	*OAS3*, *IFI44*, *CXCL10*
14	*HP*
12	*ADAM33*, *OAS2*, *IL-1RN*, *IFIT2*, *HERC5*, *RSAD2/Viperin*, *ARG1*, *ISG15*
11	*CXCL11*, *STAT2*, *TNF*, *FCER1A*, *IRF7*, *FCGR1A*, *CA1*, *STAT1*
10	*IFN*-γ, *IFIT1*, *CCL5*, *EPSTI1*, *MX2*
9	*CEACAM6*, *C3*, *CCL8*, *CXCL9*, *TRAC*, *IFI35*, *MX1*, *MPO*, *LCN2*, *OTOF*
8	*VDR*, *BPGM*, *IFI6*, *IL-10*, *ANXA3*, *OLFM4*, *SAMHD1*, *SERPING1*, *DEFA1*, *IFN-A13*, *RTP4*, *NOS2A*, *AIM2*, *Jun*, *OASL*, *GBP4*
7	*SCGB1A1*, *ISG20*, *CHI3L1*, *OAS1A*, *MUC5AC*, *PRIC285*, *IL-6*
6	*IFITM3*, *IL-20*, *MMP8*, *DEFA4*, *OASL2*, *ATF3*, *CXCL2*, *TMC5*, *TF*, *HBBP1*, *FCGR1B*, *IFIH1*, *CXCL8*, *CCL4*, *IL-15*, *PRF1*, *ALAS2*, *NFKBIA*, *MSP*, *ELA2*, *KLRD1*, *IL-7*, *MMP9*, *CD14*
5	*DEFA3*, *THOC4*, *CEACAM8*, *IFIT5*, *LAMP3*, *ERAF*, *IFN*-α, *ALDH1A1*, *LGALS9*, *GZMB*, *LTF*, *CCL7*, *HBM*, *OAS1*, *CTNNAL1*, *WARS*, *LY6E*, *HBD*, *IGTP*, *S100A12*, *PSMB8*, *DHX58*, *IFI1*, *IFI47*, *CCL2*, *PSMB9*, *GNLY*
4	*EIF2AK2*, *MYD88*, *BATF2*, *CMPK2*, *GMPR*, *LILRB4*, *OSMR*, *IIGP1 TNFSF13B*, *DAXX*, *HLA-G*, *HSPA8*, *IL-18BP*, *NMI*, *HLA-B*, *SAMD9L*, *CD177*, *IIGP2*, *LAP3*, *GBP2*, *USP18*, *PLAC8*, *MS4A6D*, *FCGR1*, *IFITM1*, *PARP9*, *AIF1*, *SERPINA3G*, *IFI202B*

a Genes were collated from multiple studies of RSV. A cutoff of a 2-fold increase in expression, compared to the reference group in the study from which the data were collated, was used when available. Genes were weighted as follows on the basis of the studies from which they were collated: human genetic studies, 4; human *in vivo* microarray studies, 3; human *in vitro* microarray studies, 2; mouse studies, 1. After weighting, genes were analyzed for multiple hits by a custom Perl script.

**TABLE 2:** Genes most frequently downregulated following RSV infection^a^

Weighted score	Gene(s)
9	*CLC*
6	*NDUFS1*, *PFDN5*
5	*RTN1*, *CAT*, *FCER1A*, *TSPAN8*, *ALOX15*, *GPR56*, *KLRB1*
4	*XRCC5*, *LMNA*, *UBD*, *CCT3*, *HSPA8*, *GARS*

a Genes were collated from multiple studies of RSV. A cutoff of a 2-fold decrease in expression was used when available. Genes were weighted as follows on the basis of the studies from which they were collated: human genetic studies, 4; human *in vivo* microarray studies, 3; human *in vitro* microarray studies, 2; mouse studies, 1. After weighting, genes were analyzed for multiple hits by a custom Perl script.

10.1128/mSystems.00051-16.3 Table S2  Unweighted analysis of upregulated genes. Genes were collated from multiple studies of RSV. A 2-fold increase in expression compared to the reference group in the study from which the data were collated was used as the cutoff, when available. Genes were analyzed for multiple hits by a custom Perl script. Download Table S2, DOCX file, 0.01 MB. Copyright © 2016 McDonald et al. 2016 McDonald et al. This content is distributed under the terms of the Creative Commons Attribution 4.0 International license.

10.1128/mSystems.00051-16.4 Table S3  Unweighted analysis of downregulated genes. Genes were collated from multiple studies of RSV. A 2-fold decrease in expression compared to reference group in the study from which the data were collated was used as the cutoff, when available. Genes were analyzed for multiple hits by a custom Perl script. Download Table S3, DOCX file, 0.01 MB. Copyright © 2016 McDonald et al. 2016 McDonald et al. This content is distributed under the terms of the Creative Commons Attribution 4.0 International license.

**FIG 2:**
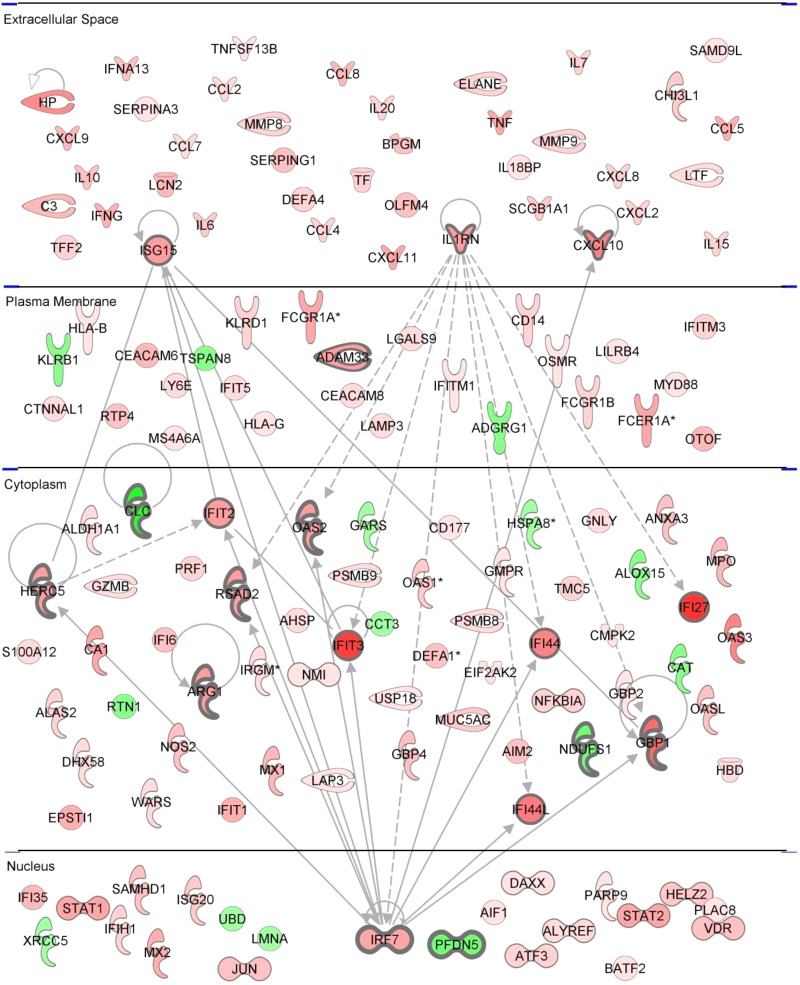
Pathway analysis of genes upregulated after RSV infection. The top 130 genes identified by a literature search are organized on the basis of their predicted functions and subcellular locations. Top candidates are indicated by bold outlining, upregulated genes are red, and downregulated genes are green. Interactions between the top 16 upregulated genes and the top 5 downregulated genes are based on known interactions in the IPA knowledge base.

To visualize the genes and interactions, we plotted the list by using the Ingenuity software platform (Fig. 2). Genes with scores of ≥6 were included. The analysis identified a mixture of secreted factors (cytokines and chemokines) and intracellular factors (transcription factors and IFN-stimulated genes [ISGs]). On the basis of our previous experience and the published literature, it was interesting that extracellular proteins upregulated in response to RSV are often associated with enhanced disease (15, 23–25), whereas intracellular proteins are associated with disease control (26). To simplify the presentation of the interactions, we focused on the interactions of the top 16 upregulated genes and the top 5 downregulated genes, looking at direct interactions that have been observed experimentally. The main observation from this was that *IRF7*, which was found to be upregulated in nine independent studies (7, 27–32), interacted with several of the other most commonly identified genes. *IRF7* was also central when the same data were analyzed for canonical pathways: overall, the genes tended to fall into pathways associated with the inflammatory response to viral infection (Table 3).

**TABLE 3:** Canonical pathways*^a^*

Ingenuity canonical pathway	Molecules
IFN signaling	OAS1, IFIT1, IFN-γ, IFITM1, STAT1, IFN-A1/IFN-A13, IFIT3, STAT2, MX1, IFI35, IFITM3, PSMB8
Activation of IRF by cytosolic pattern recognition receptors	Jun, DHX58, STAT2, IFIT2, IL-6, NFKBIA, IRF7, STAT1, TNF, IFN-A1/IFN-A13, ISG15, IFIH1, IL-10
Communication between innate and adaptive immune cells	IL-15, TNFSF13B, IFN-γ, CCL5, CXCL10, HLA-G, IL-6, CXCL8, IL-1RN, TNF, IFN-A1/IFN-A13, IL-10, HLA-B, CCL4
Role of hypercytokinemia/hyperchemokinemia in pathogenesis of influenza	IL-15, IL-1RN, IFN-γ, CCL5, CXCL10, TNF, IFN-A1/IFN-A13, CCL2, CCL4, IL-6, CXCL8
Role of pattern recognition receptors in recognition of bacteria and viruses	EIF2AK2, OAS1, IFN-γ, C3, MYD88, CCL5, OAS2, IL-6, CXCL8, IRF7, TNF, IFN-A1/IFN-A13, OAS3, IFIH1, IL-10
Granulocyte adhesion and diapedesis	CXCL9, CCL8, CCL7, CCL5, MMP9, CXCL10, CXCL2, CXCL8, CXCL11, IL-1RN, TNF, CCL2, MMP8, CCL4
Agranulocyte adhesion and diapedesis	CXCL9, CCL8, CCL7, CCL5, MMP9, CXCL10, CXCL2, CXCL8, CXCL11, IL-1RN, TNF, CCL2, MMP8, CCL4
Role of cytokines in mediating communication between immune cells	IL-15, IL-1RN, IFN-γ, TNF, IFN-A1/IFN-A13, IL-20, IL-10, IL-6, CXCL8
Differential regulation of cytokine production in intestinal epithelial cells by IL-17A and IL-17F	IFN-γ, CCL5, TNF, CCL2, IL-10, CCL4, LCN2
Dendritic cell maturation	FCGR1B, IL-15, MYD88, FCGR1A, STAT2, IL-6, NFKBIA, IL-1RN, STAT1, TNF, IFN-A1/IFN-A13, IL-10, HLA-B

a Ingenuity pathway analysis was applied to the top-scoring genes.

### Clinical validation of the bioinformatic list.

**FIG 3:**
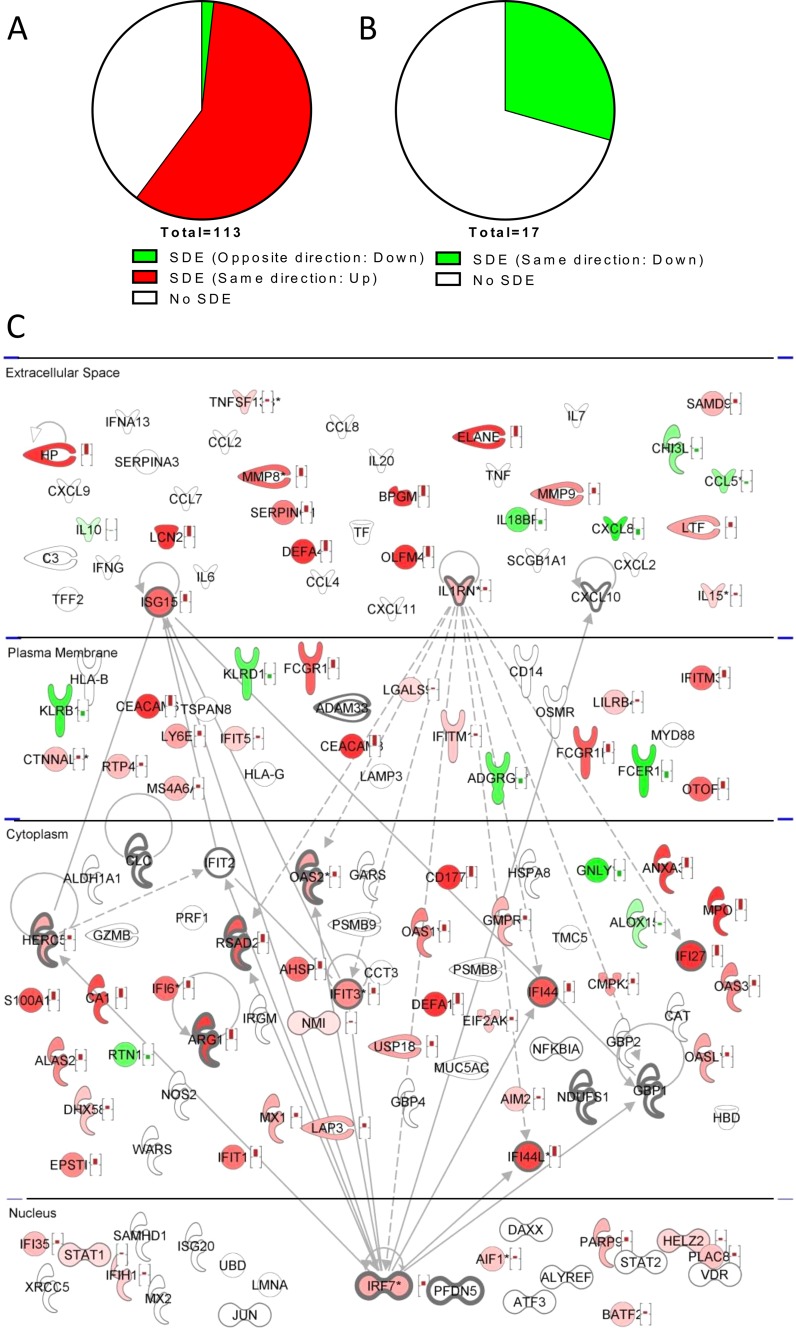
Validation of bioinformatic screening of a patient cohort. SDE genes from the clinical cohort were compared with the literature-derived gene list. Shown are overlaps between the gene lists expressed as pie charts, with directionality of agreement indicated for genes on the literature-derived list that are upregulated (A) or downregulated (B). Also shown are relative expression data from RSV-infected patients overlaid on the gene network derived from the literature-derived list (C). Upregulated genes are red, downregulated genes are green, genes identified in bioinformatic study but not clinical study are white, shading represents differential expression, and genes on the literature-derived list are indicated by bold outlining.

Since the list of candidate genes was generated by a literature-mining approach, we sought validation by using whole blood gene expression data from patients infected with RSV. We compared the transcriptomic profile of children hospitalized because of infection with RSV to that of age-matched healthy controls by using microarrays. The list of genes derived from the literature (Table 1) was compared to the genes that were significantly differentially expressed (SDE) in children infected with RSV versus healthy controls, and an overlap of 73 out of 130 genes was observed. Of the genes identified as upregulated in the literature-derived list, 66 were observed to be significantly upregulated in the clinical study, 45 were not SDE, and only 2 were SDE but in the opposite direction (Fig. 3A). The list of downregulated gene from the literature was smaller but also had a smaller proportion of agreement with the clinical study—only 5 of 17 genes were present in both studies (Fig. 3B). These data were then mapped onto the network built by using the literature-derived list (Fig. 3C), demonstrating that many of the top-ranking genes from the literature-derived list, including *IRF7*, *OAS2*, *RSAD2*, *HERC5*, *ISG15*, *IFI44*, *IL-1RN*, *ARG1*, and *IFIT3*, were in agreement between the two methods.

### Validation of the gene list by *in vitro* assay.

**FIG 4:**
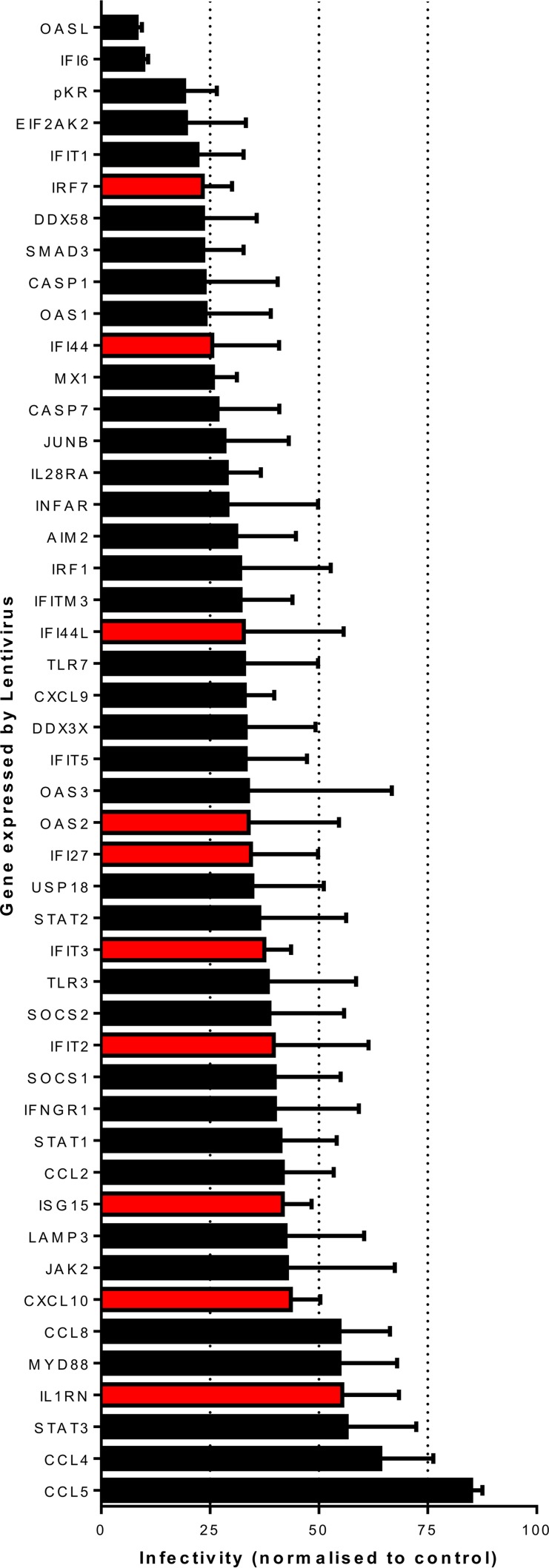
Flow cytometry confirmation of inhibitory functions of genes identified *in silico*. HEp-2 cells were transduced with lentiviral vectors expressing genes of interest identified by *in silico* screening, and 24 h later, the cells were infected with RSV expressing GFP. Cells were harvested at 48 h postinfection, and expression relative to that of control lentivirus-transfected wells was assessed. Each bar represents the mean value of three experiments ± the standard error of the mean. Red bars represent the top upregulated genes on the literature-derived list.

Of note, a number of the genes that we identified as commonly upregulated have no known role in the control of RSV infection. We wished to screen the genes identified for their effects on RSV infection by the flow cytometry-based screening method described by Schoggins et al. (7). We screened 39 ISGs identified by our *in silico* screening, and we also included receptors and transcription factors identified as upstream regulators by pathway analysis in previous studies (*IL-28RA*, *IRF1*, *IRF2*, *SOCS1*, *SOCS2*, *STAT3*, *TLR3*, and *TLR7*). The HEp-2 epithelial cell line was used, as it represents the cell lineages that RSV first encounters during an infection. HEp-2 cells were transduced with lentiviral vectors expressing each ISG and red fluorescent protein (RFP) in the same vector prior to infection with RSV expressing green fluorescent protein (GFP) (33). PKR, IFI6, and OASL overexpression reduced the GFP expression level (and therefore infection) by more than 75% of the control (Fig. 4). Of the genes identified with the most hits in the *in silico* studies (score of >12), the reductions in infectivity were as follows: *IFI27*, 65.6% ± 26.6%; *IFIT3*, 62.5% ± 10.6%; *IFI44L*, 67.3% ± 39.7%; *GBP1*, not in panel; *OAS3*, 66.3% ± 36.0%; *IFI44*, 74.5% ± 25.5%; *ISG15*, 58.3% ± 11.5%. *IRF7* overexpression led to a 76.7% ± 11.7% reduction in RSV replication, which demonstrates that, in addition to being centrally located in the predicted gene networks from *in silico* analysis, *IRF7* has a role in the control of RSV infection. The *in vitro* data support a role in viral control for the genes identified by our novel screening method.

### Validation of the gene list by an *in vivo* infection model.

**FIG 5:**
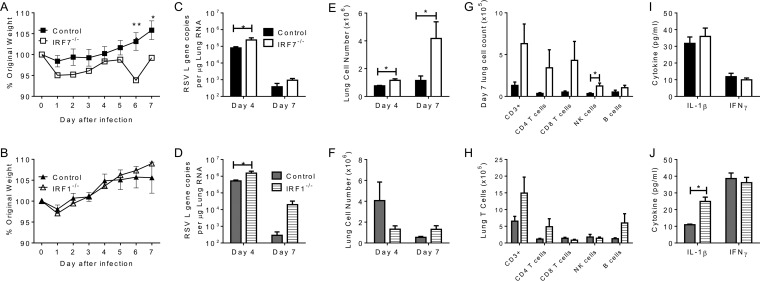
IRF7, but not IRF1, is important in the control of RSV infection. *IRF7^−/−^* (A, C, E, G, I) or *IRF1^−/−^* (B, D, F, H, I) mice were infected with 5 × 10^5^ PFU of RSV strain A2 and compared to wild-type controls on the same background. Mice were weighed daily, and weight changes were recorded as percentages of the original weight (A, F). Lungs were excised, and viral loads were calculated by quantitative PCR on days 4 and 7 postinfection (B, G). Total lung cell counts (C, H) were calculated, along with the total numbers of CD3, CD4, and CD8 (T); CD19 (B); and DX5^+^ (NK) (D, I) cells measured in the lungs by flow cytometry on day 7 postinfection. Levels of the inflammatory cytokines IL-1β and IFN-γ in the lungs (E, J) were measured by enzyme-linked immunosorbent assay on day 7 postinfection. Results are mean values ± the standard error of the mean (*n* >5). Statistical significance was assessed by Student *t* test (*, *P* < 0.05; **, *P* < 0.01; ***, *P* < 0.001).

The overarching aim of this study was to identify new genes of interest for further study. The informatic and *in vitro* analyses identified *IRF7* as being involved in the response to RSV, and it has not previously been studied in the context of RSV infection. To validate our screening approach, we compared RSV infection in mice deficient in *IRF7*, a gene on our list, with one that is associated with antiviral responses but is not on our list (*IRF1*). *IRF7^−/−^* and *IRF1*^−/−^ mice were intranasally infected with 5 × 10^5^ PFU of RSV-A (A2 strain) and monitored daily for weight loss for 7 days postinfection. Cohorts of mice were sacrificed on days 4 and 7 postinfection to quantify viral burdens and immunological changes over the course of the challenge. Mice were compared to wild-type controls on the same background. *IRF7*^−/−^ mice showed significant weight loss on days 6 and 7 postinfection compared to their wild-type littermates (*P* < 0.01) (Fig. 5A). There was no difference in weight loss between *IRF1*^−/−^ and wild-type controls (Fig. 5B). RSV loads were significantly greater in both *IRF7^−/−^* and *IRF1*^−/−^ mice on day 4 postinfection (*P* < 0.05, Fig. 5C and D) but not on day 7 postinfection. Cellular infiltrate was quantified over the course of infection, which showed a significant increase in total cells resident in the lungs on day 7 postinfection in *IRF7*^−/−^ mice but not in *IRF1*^−/−^ mice (*P* < 0.05, Fig. 5E and F). Flow cytometry revealed an increase in all cellular subpopulations in *IRF7*^−/−^ mice relative to those of wild-type mice on day 7 postinfection. In particular, the total numbers of NK cells in the lungs were significantly higher (*P* < 0.05) (Fig. 5G and H); there was no significant difference in the *IRF1^−/−^* mice. Analysis of inflammatory cytokines present in the lungs revealed differences on day 7 postinfection (Fig. 5I and J), with significantly higher levels of IL-1β in the lungs of *IRF1*^−/−^ mice than in those of wild-type controls (*P* < 0.05).

## DISCUSSION

Here we used a novel integrative approach to identify and characterize genes that are upregulated in response to RSV infection for further analysis. Previous studies have explored genetic signatures to discriminate RSV infection from other viral infections (21, 22). Our approach enabled the identification of relevant genes in a hypothesis-free fashion, identifying genes with both known and unknown functions. We used a novel algorithm that permitted the integration of multiple large genetic, gene expression, and protein data sets to identify genes consistently upregulated after RSV infection across multiple model systems. Using this approach, we were able to distill multiple heterogeneous studies into a single list of candidate molecules, generate testable hypotheses, and then demonstrate functional relevance. While we focused on RSV, this approach is broadly applicable to other pathogens for which large sets of gene expression data are available, and the data mining program is available as an open-source program.

We identified a number of genes with no known function in RSV disease as potential targets for future investigation, including *IFI27*, *IFIT3*, *IFI44L*, *GBP1*, *OAS3*, and *IFI4*4. Several of these have reported roles from other infections, but a role in RSV infection has not been reported. IFI27 (also called ISG12) plays a proinflammatory role by inducing the nuclear export of an anti-inflammatory nuclear receptor, NR4A1 (34), and recently has been related to proliferation and human epidermal cell cycling (35); GBP1 is a GTPase with a possible role in actin remodeling (36); IFI44 is antiproliferative; OAS3 interacts with RNase L (37); and ISG15 is a ubiquitin-like modifier (38) that has been shown to reduce RSV viral growth *in vitro* (39). However, some of the most frequently upregulated genes, for example, *IFI44L*, have no assigned molecular function. We took some of these genes forward into an *in vitro* assay and observed that there was a partial reduction of viral replication with all of the top hits tested (*GBP1* was not tested, as it was not in the lentiviral panel). We focused on IRF7 for the *in vivo* studies because it gave a strong knockdown *in vitro* and was central to the predicted *in silico* network. IRF7 is an amplificatory molecule responding to pattern recognition receptor detection of viral infection, inducing a further cascade of IFNs (40), and is identified as the master regulator of type I IFN-dependent immune responses (41). Previous studies have shown a role for IRF7 in human metapneumovirus (42) and influenza virus (43) infections. A recent study has demonstrated a role for IRF7 in the upregulation of RIG-I in response to RSV infection *in vitro* (44), but the present study is the first to demonstrate a central role for IRF7 in the control of RSV infection both *in vivo* and *in vitro*.

There are some limitations to this study. First, expression profiling of cells in the peripheral blood has limitations in terms of representativeness of responses in the respiratory epithelium, which RSV infects, and most of the studies are based on peripheral blood signatures. Because of the heterogeneous nature of the available databases and publications about genes associated with RSV infection, we had to make inclusion decisions that led to a slight skewing of the gene list. Where multiple gene sets were available, we chose to include genes upregulated at 24 h after infection, which may have skewed the gene set to the IFN-α/β response. Where reported, a cutoff change of 2-fold was used, but these data were not reported in several studies; likewise, not all published data had gene lists that could be incorporated into the present study. We chose a system to weight the data for the analysis giving priority to genetic association studies and *in vivo* human data over *in vitro* and mouse data. This weighting score was based on a subjective decision about the perception of the relevance of different data types to human infection. While it oversimplifies the differences both between and within different study types, once the data have been collated, other scoring systems could easily be applied to the same metadata because of the simplicity of the analysis tools used. It is of note that weighted and unweighted analyses gave similar lists. This demonstrates the power of this tool, because it can be adapted to different questions, integrating heterogeneous data sets; furthermore, the simplicity of the approach means that this can be performed quickly and easily. The *in vitro* screening contained only ISGs, which restricted the analysis of genes that were identified but not in this family; for example, GBP1. Finally, there was only a limited disease phenotype in the control mice because they were C57BL/6 mice, which are relatively resistant to RSV infection; these mice were used to match the gene knockout animals. One limiting factor in the data mining, in our experience, is a lack of standardization of published data sets; different papers have gene and protein lists in different file formats with different nomenclatures, and many only had lists in tables. Therefore, transcription of the data had to be done by hand. A more uniform approach to these data sets would enable more studies to be included in meta-analyses.

Understanding more about the functions of the genes that are most commonly upregulated following RSV infection may give us insight into pathways to control disease after infection. A number of papers have proposed genetic signatures of RSV infection; here, we show that the genes that are commonly upregulated as a result of RSV infection are characteristic of inflammation and viral control. There is an ongoing debate as to whether inflammation or virus-mediated pathology is the primary cause of disease after RSV infection. In the this context, one of the striking features of the pathway analysis was a divergence in the effects on disease outcome of the gene products localized to the extracellular space and those found in the nucleus and cytoplasm. Animal studies suggest that the extracellular proteins enhance disease following RSV infection (45) by increasing inflammation, while the intracellular proteins reduce disease by decreasing viral replication. In support of this, we have recently shown that antiviral gene *IFITM3* is important in the prevention of RSV infection (26, 46). In the *in vitro* studies, overexpression of *IFITM3* led to a 68% ± 20.6% reduction in RSV replication. Furthermore, overexpression of the chemokines CCL4, CCL5, and CCL8 had little effect on viral replication. This suggests that boosting the antiviral response without increasing inflammation would be a good strategy to control RSV disease. One potential target to achieve this would be the type I IFN response, which, if boosted, may increase the transcription of antiviral genes. However, recent studies have shown that in RSV (47) and influenza virus (48) infections, type I IFN contributes to inflammation and disease after viral infection, suggesting that there is a sweet spot of IFN production and either too little or too much can lead to a disease state. Targeting the host response may be particularly beneficial, as it is not necessarily specific to the pathogen and is less likely to induce antimicrobial resistance.

There are several other approaches for integrating heterogeneous data for immunology research (49). Selection of the tool to use depends on the desired outcome, ranging from analysis of T cell receptor and antibody repertoire analysis to network analysis and visualization. Some tools enable the integration of gene expression data with DNA variation (eQTL) (50) or against the epigenetic status of the same gene (51), both of which enable greater understanding of the processes underpinning gene regulation and expression in the immune response. For higher-level analysis, data can be merged by using network analysis tools to see novel interactions between genes (52). These are sophisticated models underpinned by statistical techniques requiring specialized skill sets and data analysis software to be performed rigorously. Our approach is not. It requires no specialist informatic skills or software (for a how-to guide, see Text S1 in the supplemental material); it uses Excel (but would work with any spreadsheet program) and a free Perl script. Additionally, it performs a different function prioritizing genes for further study. This tool generates a hypothesis-free candidate list that can be investigated further. That the genes were biologically plausible and affected RSV infection *in vitro* and *in vivo* validities it as a downselection tool. In conclusion, here we describe a novel literature data mining approach for candidate gene prioritization. It has the benefit of simplicity and is broadly applicable to a range of infectious diseases.

10.1128/mSystems.00051-16.1 Text S1  Try this at home: a step-by-step guide to using our analysis approach. Download Text S1, DOCX file, 0.01 MB. Copyright © 2016 McDonald et al. 2016 McDonald et al. This content is distributed under the terms of the Creative Commons Attribution 4.0 International license.

## MATERIALS AND METHODS

### Meta-analysis of papers and *in silico* analysis.

We selected papers published prior to July 2015 by using the search terms “RSV AND Microarray OR transcriptome OR genetic or proteome” in PubMed; other studies from previous literature searches were also included (see Table S1 in the supplemental material). Papers were not included if the data set described in the study with fold change and time point data were not easily accessible. Individual genes were included for analysis if they were reported to have a >2-fold difference from the reference group in the specific study. Gene lists were harvested from the published literature and collated in a single excel file. For analysis, genes were given subjective weighting scores based on the types of studies from which they were collated, as follows: human genetic studies, 4; human *in vivo* transcriptomic studies, 3; human *in vitro* studies, 2; murine studies, 1. A custom Perl script, countIDs (available at https://sourceforge.net/projects/countids/), was written to parse the gene list file generated to assign a weighted score to each gene, generating an output gene list. Figure 1 is a flowchart of the process; for a step-by-step guide to the approach, see Text S1 in the supplemental material. For visualization, genes with assigned scores of ≥6 were included for Ingenuity Pathway Analysis (IPA; Qiagen).

### Validation in a clinical cohort.

We established a case-control group comprising 27 RSV patients and 80 healthy controls. Whole blood (2.5 ml) was collected into PAXgene blood RNA tubes (PreAnalytiX; Germany), incubated for 2 h, frozen at −20°C within 6 h of collection, and then stored at −80°C. RNA was extracted with PAXgene blood RNA kits (PreAnalytiX; Germany) according to the manufacturer’s instructions. The integrity and yield of the total RNA were assessed with an Agilent 2100 Bioanalyzer and a NanoDrop 1000 spectrophotometer. After quantification and quality control, biotin-labeled cRNA was prepared with Illumina TotalPrep RNA amplification kits (Applied Biosystems) from 500 ng of RNA. Labeled cRNA was hybridized overnight to HumanHT-12 v4 Expression BeadChip arrays (Illumina). After washing, blocking, and staining, the arrays were scanned with an Illumina BeadArray Reader according to the manufacturer’s instructions. With Genome Studio software, the microarray images were inspected for artifacts and quality control parameters were assessed. Data were analyzed in the R language and environment for statistical computing (version 3.1.2) (53). Mean raw intensity values for each transcript were transformed to a logarithmic scale (base 2), corrected for local background intensities, and normalized by robust spline normalization. We identified the transcripts that were SDE between the RSV-infected children and the healthy control group with an adjusted *P* value of <0.05 by using a linear model for transcript expression. The functions lmFit and eBayes in the R package limma were used to calculate statistics.

### Validation *in vitro.*

ISG library creation and screening were performed with a modified version of the assay described previously (7). A total of 2 × 10^5^ HEp-2 cells was seeded into 96-well plates overnight prior to transfection with 10^5^ of the individual RFP-ISG lentiviruses in Dulbecco’s modified Eagle’s medium supplemented with 20 mM HEPES and 4 mg/ml Polybrene by spinoculation at 1,000 rpm for 1 h. Twenty-four hours later, the cells were infected with GFP-expressing RSV (RSV-GFP) (33), and 24 h after that, the cells were harvested for analysis by flow cytometry. Live/dead discrimination was performed by addition of the LIVE/DEAD Fixable Aqua Dead Cell Stain kit (Molecular Probes) prior to the acquisition of data on an LSRFortessa (BD). Viral infection was determined on the basis of the percentage of GFP-RFP double-positive cells, and the relative infectivity in each well was normalized to the samples transfected with control lentivirus only. Data were analyzed with CyAn ADP Summit 4.3.

### Validation *in vivo* with gene knockout mice.

Sex-matched 8- to 10-week-old wild-type C57BL/6N *IRF1*^tm1a(*EUCOMM*)*Wtsi*^ and *IRF7*^tm1(*KOMP*)*Wtsi*^ mice (54) (Wellcome Trust Sanger Institute) were maintained in accordance with United Kingdom Home Office regulations and United Kingdom Animals (Scientific Procedures) Act 1986 under project license PPL 80/2596. Animals were supplied with food and water *ad libitum* and monitored daily for signs of illness. Founder mice were phenotyped through pipelines at the Wellcome Trust Sanger Institute as described previously (9, 10).

### RSV *in vivo* studies.

RSV strain A2 (from P. Openshaw, Imperial College London) was grown in HEp-2 cells, and viral titers were determined by plaque assay. Mice were infected intranasally with 5 × 10^5^ PFU in a volume of 100 µl under isoflurane anesthesia. They were weighed daily to monitor disease severity. Their lungs were removed, the smaller lobe was snap-frozen in liquid nitrogen for RNA extraction, and the remainder was homogenized by passage through 100-µm cell strainers (Falcon). Red blood cells in the lung sample were lysed in ammonium chloride buffer, and the remaining cells were resuspended in RPMI medium with 10% fetal calf serum. Viable cell numbers were determined by trypan blue exclusion, and lung cell types were differentiated by flow cytometry as described previously (55). In brief, cells were suspended in Fc block (anti-CD16/32; BD) in phosphate-buffered saline–1% bovine serum albumin and stained with surface antibodies CD3-fluorescein isothiocyanate (BD, Oxford United Kingdom), CD4-allophycocyanin (APC; BD), CD8-APC Alexa 750 (Invitrogen, Paisley, United Kingdom), NK1.1-peridinin chlorophyll protein-Cy5.5 (BD), and CD19-eFluor 450 (eBioscience, Hatfield, United Kingdom). Cells were run on a BD FACS Aria II. Singlet lymphocyte cells were defined on the basis of their size, side scatter, and doublet discrimination, and then their immune phenotypes were analyzed on the basis of cell surface markers. RSV loads were measured by quantitative reverse transcription-PCR for the RSV L gene with primers and probes previously described (19), and L gene copy numbers were determined with an RSV L gene standard and expressed relative to the amount (micrograms) of lung RNA. The cytokines interleukin-1β (IL-1β) and IFN-γ in the lungs were quantified with DuoSet kits from R&D Systems.

### Statistical analysis.

Statistical analysis was performed with weighted Student *t* tests in GraphPad Prism 6.0.

### Microarray data accession number.

The microarray data from the clinical study have been uploaded to the Gene Expression Omnibus database (http://www.ncbi.nlm.nih.gov/geo/) and assigned accession number GSE80179.
