# Supplementary material for: A Simple Screening Approach To Prioritize Genes for Functional Analysis Identifies a Role for Interferon Regulatory Factor 7 in the Control of Respiratory Syncytial Virus Disease
Source: mSystems. 2016 Jun 28;1(3):e00051-16. doi: 10.1128/mSystems.00051-16 (PMC5069771; doi:10.1128/mSystems.00051-16)
Supplement: Table S1 [file sys003162034st2.docx]

Table S1. Datasets mined for study. Papers published prior to July 2015 were selected, using the search terms “RSV AND Microarray OR transcriptome OR genetic or proteome” on PubMed. Table incorporates subjective weighting score based on the type of study they were collated from (in brackets): human genetic studies (4), human in vivo transcriptomic studies (3), human in vitro studies (2), murine studies (1). Sample type, time point and analysis method also included.

| **Weighting** | **Human or Mouse** | **Sample** | **Patient Description** | **Time Point included** | **Analysis** | **Reference/ PMID** |
| --- | --- | --- | --- | --- | --- | --- |
| 4 | Human | Meta-analysis reviews | RSV severity in patients | N/A | Genetics/ GWAS | (1, 2) |
| 4 | Human | Whole Blood or buccal swabs | Hospitalized for RSV bronchiolitis and parents | N/A | DNA: Beadarray technology (Illumina) (SNP) | (3) |
| 4 | Human | Whole Blood or buccal swabs | Same cohort as above focusing on preterm children | N/A | DNA- Beadarray technology (Illumina) (SNP) | (4) |
| 4 | Human | Whole Blood or buccal swabs | Hospitalized RSV lower respiratory tract infection | N/A | DNA- Beadarray technology (Illumina) (SNP) | (5) |
| 3 | Human | Whole Blood | Hospitalized RSV bronchiolitis |  | RNA- microarray | (6) |
| 3 | Human | PBMC | Hospitalized RSV bronchiolitis, graded for severity |  | RNA- microarray | (7) |
| 3 | Human | Cord blood | Hospitalized with RSV bronchiolitis Vs. control. | N/A | RNA- microarray | (8) |
| 3 | Human | Whole Blood | Symptomatic Adults RSV Vs. Asymptomatic | Peak Symptoms | RNA- microarray | (9) |
| 3 | Human | Whole Blood | Hospitalized RSV bronchiolitis | N/A | RNA- microarray | (10) |
| 3 | Human | Whole Blood | Hospitalized RSV bronchiolitis | N/A | RNA- microarray | (11) |
| 3 | Human | Whole Blood | Hospitalized RSV bronchiolitis | N/A | RNA- microarray | (12) |
| 3 | Human | Nasal mucosa cytology scrape | Hospitalized RSV | N/A | miRNA- microarray | (13) |
| 3 | Human | Nasopharyngeal aspirates | Hospitalized RSV lower respiratory tract infection | N/A | RNA- microarray | (14) |
| 3 | Human | Nasopharyngeal aspirates | Hospitalized RSV | N/A | Proteomics | (15) |
| 2 | Human | Airway epithelial cells | Hospitalized with acute RSV | 48 hrs post infection | RNA- microarray | (16) |
| 2 | Human | Human bronchial epithelial cells | N/A | 24 hrs post infection | RNA- microarray | (17) |
| 2 | Human | SPC-A1 cells (Human type II alveolar cell line) | N/A | 24 hrs post infection | RNA-differential display RT-PCR | (18) |
| 2 | Human | A549 cells (Human epithelial cell line) | N/A | 24 hrs post infection | RNA- microarray | (19) |
| 2 | Human | HEp2 Cells | N/A | 48 hrs post infection | RNA- microarray | (20) |
| 2 | Human | HeLa Cells | N/A | N/A | ISG overexpression | (21) |
| 2 | Human | A549 Cells | N/A | 6,12 or 24 | Proteomics | (22) |
| 2 | Human | HEp2 cells | N/A | 24 hrs post infection | Protein- UPLC-MSE analysis, Western Blot & qRT-PCR | (23) |
| 1 | Virus: Human | Chip | N/A |  | Microfluidics – Protein:Protein | (24) |
| 1 | Virus Human | Viral Preps | N/A |  | Proteomics | (25) |
| 1 | Mouse | Lung Tissue | Female specific-pathogen-free BALB/c mice, aged 6 – 10 weeks | 24 hrs post infection | RNA- microarray | (26) |
| 1 | Mouse | Lung, bronchial lymph node and blood | BALB/c mice of 6–10 weeks of age | 24 hrs post infection | RNA- microarray | (27) |
| 1 | Mouse | Lung Tissue | Female specific-pathogen-free BALB/c mice, aged 6 – 10 weeks | 24 hrs post infection | RNA- microarray | (28) |
| 1 | Mouse | Lung Tissue | Male BALB/c mice, aged 6–8 weeks, weighing 21–28 g | 5 days post infection | RNA- microarray | (29) |
| 1 | Mouse | Lung Tissue | Female C57BL/6J and AKR/J mice at 6 to 8 weeks of age | 24 hrs post infection | RNA- microarray | (30) |
| 1 | Mouse | Murine lung macrophages | Female special pathogen-free BALB/c mice 6 – 8 weeks old | 24 hrs post infection | RNA- microarray | (31) |
| 1 | Mouse | Lung Tissue | Old (19–21 mos) and young (2–3 mos) BALB/c mice | 24 hrs post infection | RNA- RT2-PCR Profiler Array | (32) |
| 1 | Mouse | Lung Tissue | Aged mice (>10 mos) and sham BALB/c | 4 days post infection | RNA- microarray | (33) |

**References**

1. **Tregoning JS, Schwarze J.** 2010. Respiratory viral infections in infants: causes, clinical symptoms, virology, and immunology. Clin Microbiol Rev **23:**74-98.

2. **Miyairi I, DeVincenzo JP.** 2008. Human genetic factors and respiratory syncytial virus disease severity. Clin Microbiol Rev **21:**686-703.

3. **Janssen R, Bont L, Siezen CL, Hodemaekers HM, Ermers MJ, Doornbos G, van 't Slot R, Wijmenga C, Goeman JJ, Kimpen JL, van Houwelingen HC, Kimman TG, Hoebee B.** 2007. Genetic susceptibility to respiratory syncytial virus bronchiolitis is predominantly associated with innate immune genes. J Infect Dis **196:**826-834.

4. **Siezen CL, Bont L, Hodemaekers HM, Ermers MJ, Doornbos G, Van't Slot R, Wijmenga C, Houwelingen HC, Kimpen JL, Kimman TG, Hoebee B, Janssen R.** 2009. Genetic susceptibility to respiratory syncytial virus bronchiolitis in preterm children is associated with airway remodeling genes and innate immune genes. Pediatr Infect Dis J **28:**333-335.

5. **Ermers MJ, Janssen R, Onland-Moret NC, Hodemaekers HM, Rovers MM, Houben ML, Kimpen JL, Bont LJ.** 2011. IL10 family member genes IL19 and IL20 are associated with recurrent wheeze after respiratory syncytial virus bronchiolitis. Pediatr Res **70:**518-523.

6. **Fjaerli HO, Bukholm G, Krog A, Skjaeret C, Holden M, Nakstad B.** 2006. Whole blood gene expression in infants with respiratory syncytial virus bronchiolitis. BMC Infect Dis **6:**175.

7. **Brand HK, Ahout IM, de Ridder D, van Diepen A, Li Y, Zaalberg M, Andeweg A, Roeleveld N, de Groot R, Warris A, Hermans PW, Ferwerda G, Staal FJ.** 2015. Olfactomedin 4 Serves as a Marker for Disease Severity in Pediatric Respiratory Syncytial Virus (RSV) Infection. PLoS One **10:**e0131927.

8. **Fjaerli HO, Bukholm G, Skjaeret C, Holden M, Nakstad B.** 2007. Cord blood gene expression in infants hospitalized with respiratory syncytial virus bronchiolitis. J Infect Dis **196:**394-404.

9. **Zaas AK, Chen M, Varkey J, Veldman T, Hero AO, 3rd, Lucas J, Huang Y, Turner R, Gilbert A, Lambkin-Williams R, Oien NC, Nicholson B, Kingsmore S, Carin L, Woods CW, Ginsburg GS.** 2009. Gene expression signatures diagnose influenza and other symptomatic respiratory viral infections in humans. Cell Host Microbe **6:**207-217.

10. **Herberg JA, Kaforou M, Gormley S, Sumner ER, Patel S, Jones KD, Paulus S, Fink C, Martinon-Torres F, Montana G, Wright VJ, Levin M.** 2013. Transcriptomic profiling in childhood H1N1/09 influenza reveals reduced expression of protein synthesis genes. J Infect Dis **208:**1664-1668.

11. **Mejias A, Dimo B, Suarez NM, Garcia C, Suarez-Arrabal MC, Jartti T, Blankenship D, Jordan-Villegas A, Ardura MI, Xu Z, Banchereau J, Chaussabel D, Ramilo O.** 2013. Whole blood gene expression profiles to assess pathogenesis and disease severity in infants with respiratory syncytial virus infection. PLoS Med **10:**e1001549.

12. **Bucasas KL, Mian AI, Demmler-Harrison GJ, Caviness AC, Piedra PA, Franco LM, Shaw CA, Zhai Y, Wang X, Bray MS, Couch RB, Belmont JW.** 2013. Global gene expression profiling in infants with acute respiratory syncytial virus broncholitis demonstrates systemic activation of interferon signaling networks. Pediatr Infect Dis J **32:**e68-76.

13. **Inchley CS, Sonerud T, Fjaerli HO, Nakstad B.** 2015. Nasal mucosal microRNA expression in children with respiratory syncytial virus infection. BMC Infect Dis **15:**150.

14. **van den Kieboom CH, Ahout IM, Zomer A, Brand KH, de Groot R, Ferwerda G, de Jonge MI.** 2015. Nasopharyngeal gene expression, a novel approach to study the course of respiratory syncytial virus infection. Eur Respir J **45:**718-725.

15. **Fornander L, Ghafouri B, Kihlstrom E, Akerlind B, Schon T, Tagesson C, Lindahl M.** 2011. Innate immunity proteins and a new truncated form of SPLUNC1 in nasopharyngeal aspirates from infants with respiratory syncytial virus infection. Proteomics Clin Appl **5:**513-522.

16. **Ioannidis I, McNally B, Willette M, Peeples ME, Chaussabel D, Durbin JE, Ramilo O, Mejias A, Flano E.** 2012. Plasticity and virus specificity of the airway epithelial cell immune response during respiratory virus infection. J Virol **86:**5422-5436.

17. **Huang YC, Li Z, Hyseni X, Schmitt M, Devlin RB, Karoly ED, Soukup JM.** 2008. Identification of gene biomarkers for respiratory syncytial virus infection in a bronchial epithelial cell line. Genomic Med **2:**113-125.

18. **Zhao D, Peng D, Li L, Zhang Q, Zhang C.** 2008. Inhibition of G1P3 expression found in the differential display study on respiratory syncytial virus infection. Virol J **5:**114.

19. **Martinez I, Lombardia L, Garcia-Barreno B, Dominguez O, Melero JA.** 2007. Distinct gene subsets are induced at different time points after human respiratory syncytial virus infection of A549 cells. J Gen Virol **88:**570-581.

20. **Zhang W, Zhang L, Zan Y, Du N, Yang Y, Tien P.** 2015. Human respiratory syncytial virus infection is inhibited by IFN-induced transmembrane proteins. J Gen Virol **96:**170-182.

21. **Schoggins JW, MacDuff DA, Imanaka N, Gainey MD, Shrestha B, Eitson JL, Mar KB, Richardson RB, Ratushny AV, Litvak V, Dabelic R, Manicassamy B, Aitchison JD, Aderem A, Elliott RM, Garcia-Sastre A, Racaniello V, Snijder EJ, Yokoyama WM, Diamond MS, Virgin HW, Rice CM.** 2014. Pan-viral specificity of IFN-induced genes reveals new roles for cGAS in innate immunity. Nature **505:**691-695.

22. **van Diepen A, Brand HK, Sama I, Lambooy LH, van den Heuvel LP, van der Well L, Huynen M, Osterhaus AD, Andeweg AC, Hermans PW.** 2010. Quantitative proteome profiling of respiratory virus-infected lung epithelial cells. J Proteomics **73:**1680-1693.

23. **Ternette N, Wright C, Kramer HB, Altun M, Kessler BM.** 2011. Label-free quantitative proteomics reveals regulation of interferon-induced protein with tetratricopeptide repeats 3 (IFIT3) and 5'-3'-exoribonuclease 2 (XRN2) during respiratory syncytial virus infection. Virol J **8:**442.

24. **Kipper S, Hamad S, Caly L, Avrahami D, Bacharach E, Jans DA, Gerber D, Bajorek M.** 2015. New host factors important for respiratory syncytial virus (RSV) replication revealed by a novel microfluidics screen for interactors of matrix (M) protein. Mol Cell Proteomics **14:**532-543.

25. **Radhakrishnan A, Yeo D, Brown G, Myaing MZ, Iyer LR, Fleck R, Tan BH, Aitken J, Sanmun D, Tang K, Yarwood A, Brink J, Sugrue RJ.** 2010. Protein analysis of purified respiratory syncytial virus particles reveals an important role for heat shock protein 90 in virus particle assembly. Mol Cell Proteomics **9:**1829-1848.

26. **Janssen R, Pennings J, Hodemaekers H, Buisman A, van Oosten M, de Rond L, Ozturk K, Dormans J, Kimman T, Hoebee B.** 2007. Host transcription profiles upon primary respiratory syncytial virus infection. J Virol **81:**5958-5967.

27. **Pennings JL, Schuurhof A, Hodemaekers HM, Buisman A, de Rond LC, Widjojoatmodjo MN, Luytjes W, Kimpen JL, Bont L, Janssen R.** 2011. Systemic signature of the lung response to respiratory syncytial virus infection. PLoS ONE **6:**e21461.

28. **Schuurhof A, Bont L, Pennings JL, Hodemaekers HM, Wester PW, Buisman A, de Rond LC, Widjojoatmodjo MN, Luytjes W, Kimpen JL, Janssen R.** 2010. Gene expression differences in lungs of mice during secondary immune responses to respiratory syncytial virus infection. J Virol **84:**9584-9594.

29. **Hennus MP, Janssen R, Pennings JL, Hodemaekers HM, Kruijsen D, Jansen NJ, Meyaard L, van Vught AJ, Bont LJ.** 2012. Host response to mechanical ventilation for viral respiratory tract infection. Eur Respir J **40:**1508-1515.

30. **Stark JM, Barmada MM, Winterberg AV, Majumber N, Gibbons WJ, Jr., Stark MA, Sartor MA, Medvedovic M, Kolls J, Bein K, Mailaparambil B, Krueger M, Heinzmann A, Leikauf GD, Prows DR.** 2010. Genomewide association analysis of respiratory syncytial virus infection in mice. J Virol **84:**2257-2269.

31. **Ravi LI, Li L, Sutejo R, Chen H, Wong PS, Tan BH, Sugrue RJ.** 2013. A systems-based approach to analyse the host response in murine lung macrophages challenged with respiratory syncytial virus. BMC Genomics **14:**190.

32. **Wong TM, Boyapalle S, Sampayo V, Nguyen HD, Bedi R, Kamath SG, Moore ML, Mohapatra S, Mohapatra SS.** 2014. Respiratory syncytial virus (RSV) infection in elderly mice results in altered antiviral gene expression and enhanced pathology. PLoS One **9:**e88764.

33. **Mosquera RA, Stark JM, Atkins CL, Colasurdo GN, Chevalier J, Samuels CL, Pacheco SS.** 2014. Functional and immune response to respiratory syncytial virus infection in aged BALB/c mice: a search for genes determining disease severity. Exp Lung Res **40:**40-49.
