# Supplementary material for: A Simple Screening Approach To Prioritize Genes for Functional Analysis Identifies a Role for Interferon Regulatory Factor 7 in the Control of Respiratory Syncytial Virus Disease
Source: mSystems. 2016 Jun 28;1(3):e00051-16. doi: 10.1128/mSystems.00051-16 (PMC5069771; doi:10.1128/mSystems.00051-16)
Supplement: Table S2 [file sys003162034st3.docx]

Table S2 Unweighted analysis – upregulated genes. Genes were collated from multiple studies of RSV; a cut-off of a two fold increase in expression compared to reference group in the study data was collated from, was used where available. Genes were analyzed for multiple hits by a custom PERL script.

| **Number of studies gene included in** | **Gene name** |
| --- | --- |
| 14 | IFIT3 |
| 12 | IFI27 |
| 10 | GBP1 |
| 9 | CXCL10, IRF7 |
| 8 | IFIT2 |
| 7 | IFI44L, OAS2, CXCL11, OAS1A, GBP4, STAT1, IFI44, ISG15 |
| 6 | SAMHD1, OASL2, IFIT1, CXCL9, IL1RN, IFI35, HERC5, STAT2, OAS3, RSAD2, CCL4 |
| 5 | CXCL2, ISG20, CCL7, RTP4, HP, CCL5, MX1, TNF, IFIH1, ARG1, IGTP, LCN2, IFI1, IFI47, CCL2, MX2 |
| 4 | IFITM3, LILRB4, IIGP1, IFNG, CCL8, LGALS9, DAXX, IL18BP, IIGP2, FCGR1A, AIM2, GBP2, USP18, PLAC8, EPSTI1, MS4A6D, PSMB8, FCGR1, DHX58, CA1, PARP9, AIF1, SERPINA3G, IL6, IFI202B |
