# Supplementary material for: A Simple Screening Approach To Prioritize Genes for Functional Analysis Identifies a Role for Interferon Regulatory Factor 7 in the Control of Respiratory Syncytial Virus Disease
Source: mSystems. 2016 Jun 28;1(3):e00051-16. doi: 10.1128/mSystems.00051-16 (PMC5069771; doi:10.1128/mSystems.00051-16)
Supplement: Table S3 [file sys003162034st4.docx]

Table S3 Unweighted analysis – downregulated genes Genes were collated from multiple studies of RSV; a cut-off of a two fold decrease in expression compared to reference group in the study data was collated from, was used where available. Genes were analyzed for multiple hits by a custom PERL script.

| **Number of studies gene included in** | **Gene name** |
| --- | --- |
| 3 | NDUFS1, CLC |
| 2 | RTN1, CAT, FCER1A, PLUNC, XRCC5, TSPAN8, LMNA, UBD, PFDN5, CCT3, ALOX15, HSPA8, GPR56, GARS, KLRB1 |
