# Supplementary material for: A Simple Screening Approach To Prioritize Genes for Functional Analysis Identifies a Role for Interferon Regulatory Factor 7 in the Control of Respiratory Syncytial Virus Disease
Source: mSystems. 2016 Jun 28;1(3):e00051-16. doi: 10.1128/mSystems.00051-16 (PMC5069771; doi:10.1128/mSystems.00051-16)
Supplement: Text S1 [file sys003162034s1.docx]

**Figure S1. Try this at home**

1. Perform a literature search for pathogen of interest, use the terms “Microarray OR transcriptome OR genetic or proteome”.
2. Determine a cutoff for up/down regulation of genes within the published study, select a time point after infection.
3. Copy gene lists from literature found into excel. It may be necessary to convert into Gene ID (<https://david.ncifcrf.gov/conversion.jsp>) to ensure uniformity.
4. Install Perl on your Windows computer and the easiest way to do that is with ActivePerl. Go to the link to download it. There are 2 downloads and both will probably work but the safest option is probably the first "x86" version. Once downloaded run the file and follow the instructions. If you are given any options to select then the defaults should be fine. Once installed you won't need to do anything else with it. [http://www.activestate.com/activeperl/downloads#](http://www.activestate.com/activeperl/downloads)
5. Prepare the gene list as an Excel file so the script can read them. Put selected gene lists into excel in columns. Delete all of the headings but leave one blank row at the top. Next remove all of the blank columns and all of the other columns, except those with the IDs to be counted. The file should just contain all of the IDs, with the blank row at the top. The blank row is for the weighting so add the weight for each column. You'll need to add a 1 here even if there is no weighting. When complete save the file as text (Tab delimited) format.
6. Ensure the attached script is in the same location as the saved file.
7. Run the script
   1. Click Start.
   2. In the text box that says "Search programs and files" type "cmd"
   3. Click on the "cmd" icon that should appear at the top of the list and that will launch the command prompt window.
   4. Next you need to change directory to the location of the file and script. I'd recommend you save the files on the C drive in a single directory to keep it somple.
   5. Assuming the file is in a folder called "Perl", type "cd Perl".
   6. You'll now be in the correct location. To run the script you just need to add the name of the text file to analyse. Assuming it is the PrimaryUp.txt file the command is: perl countIDs.pl PrimaryUp.txt
   7. That will produce a file called PrimaryUp_count.txt.
   8. Open the count file in Excel as it is tab delimited.
